# Shigeo Satomura: 60 years of Doppler ultrasound in medicine

**DOI:** 10.1186/s12947-015-0042-3

**Published:** 2015-12-23

**Authors:** Ioan M. Coman, Bogdan A. Popescu

**Affiliations:** Department of Cardiology, University of Medicine and Pharmacy “Carol Davila”, Sos. Fundeni 258, sector 2, 022328 Bucharest, Romania; Emergency Institute of Cardiovascular Diseases “Prof. Dr. C. C. Iliescu”, Sos. Fundeni 258, sector 2, 022328 Bucharest, Romania

**Keywords:** Doppler echocardiography, Doppler, Echocardiography

## Abstract

This year we celebrate 60 years since Shigeo Satomura published the first measurements of the Doppler shift of ultrasonic signals from a beating heart. He demonstrated that Doppler signals can be retrieved from heart movements when insonated with 3 MHz ultrasonic waves. Later, togheter with Ziro Kaneko, he constructed the first Doppler flowmeter to measure the blood flow velocities in peripheral and extracranial brain-supplying vessels using ultrasounds. They proved that ultrasonic Doppler signals from arteries and veins can be recorded from the surface of the skin and pioneered transcutaneous flow analysis in systole and diastole in both normal and diseased blood vessels. These were the first medical applications of Doppler sonography and impressive technologic innovations have been continuing ever since. Over time, Doppler techniques became a key player in diagnostic ultrasound for hemodynamic assessment, replacing cardiac catheterization in many clinical settings.

## Background

Progress in medicine is tremendously linked to technological developments in other scientific fields. The use of Doppler effect in diagnostic ultrasound is an excellent example of such a translation - from physics to health science. It is worth recognizing the people who pioneered this process.

### Review

The frequency shift with emitting source movement was discovered in 1842, when the Austrian professor of mathematics and physics Christian Andreas Doppler presented the results of his investigations in the field of astronomy at the Royal Society of Sciences in Prague.

Various applications - from theories of the Universe to military sonars - were based on it but, at least until the middle of the 20th century, no one could have predicted its impact in medicine [1].

**Fig. 1:**
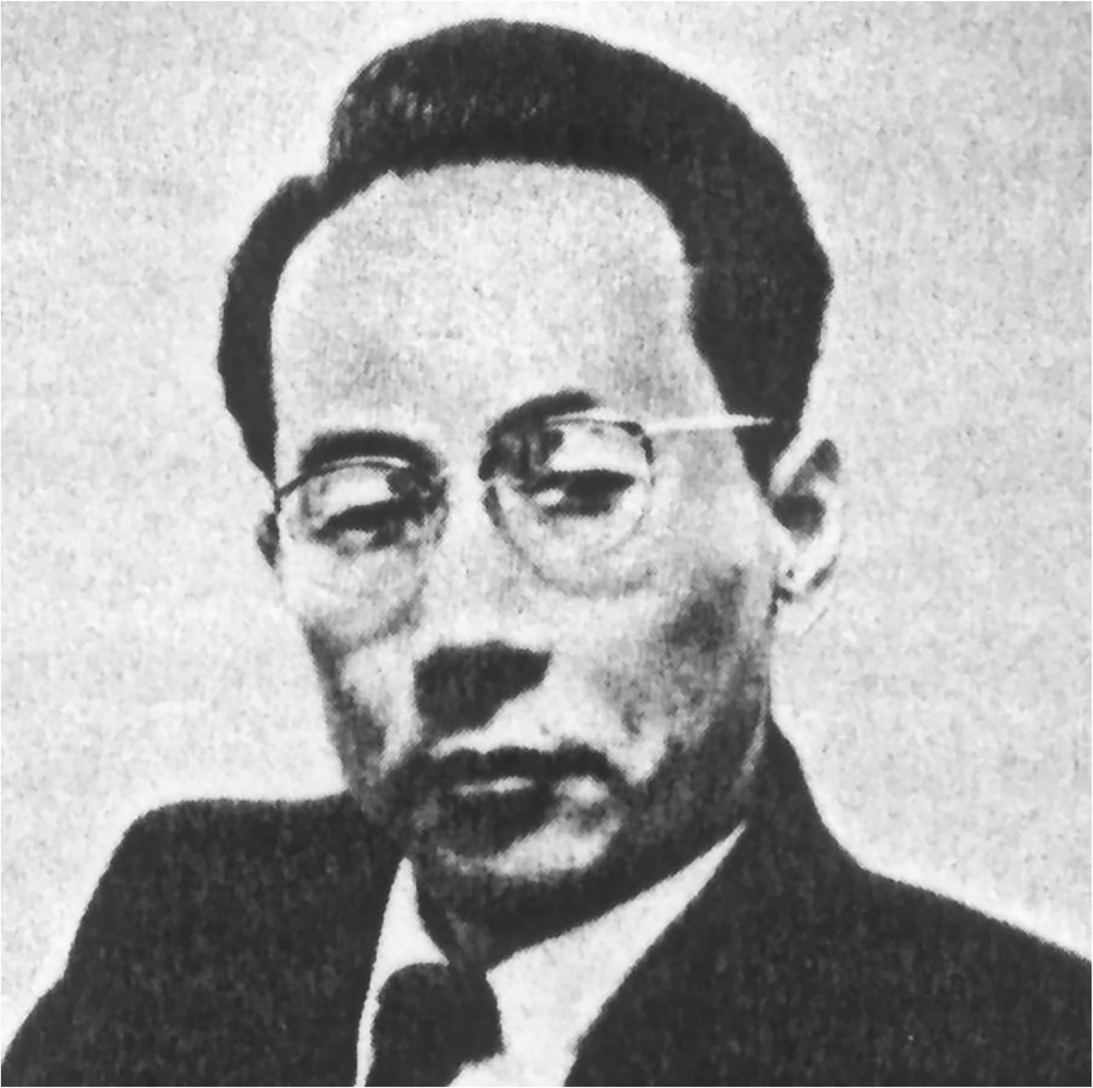
Shigeo Satomura (1919–1960). Source: Wikimedia Commons

Shigeo Satomura (Fig. 1) was born in 1919 in Osaka, Japan and attended his hometown’s School of Physics, obtaining his PhD in 1944. Thereafter, he began working at the University’s Institute of Scientific and Industrial Research where he was appointed Assistant Professor in 1952.

Among other areas of interest, he became involved in the use of radar technology with ultrasound in industrial research. His supervisor, Professor Kinjiro Okabe, suggested him to investigate the potential value of this diagnostic tool in the medical field. His new research consisted of ultrasound emission towards living bodies, recording the reflected signals and analysing their changes. The analysis was performed in accordance to Doppler’s theory using the formula f = 2u/λ, where (using authors terminology) “f is the Doppler frequency, u the velocity of the reflecting part (parallel to the ultrasonic incidence) and λ the ultrasonic wavelength in the human body”. Since the wavelength is determined by the frequency of the ultrasound used, the measurement and the analysis of the frequency of the Doppler signal provided information about the reflecting part of the body. Satomura, in collaboration with two cardiologists, T. Yoshida and Y. Nimura from the Osaka University Hospital, assessed the technique for measuring heart and (both limb and eye) blood vessels. At the end of the year, he presented and then published his first paper on the topic. The paper, entitled “A new method of the mechanical vibration measurement and its application”, showed the display of heart movements when insonated with 3 MHz ultrasonic waves. From the parasternal approach, Doppler signals of about 100 Hz were obtained in presystole, systole, and early diastole, indicating atrial contraction, ventricular contraction, and ventricular relaxation, respectively [2].

The following year this same team published the paper “Ultrasonic Doppler method for the inspection of cardiac function” were they described the prototype Doppler flowmeter. Using a low-pass filter and a band-pass filter added to the Doppler frequencies amplifier it was possible to distinguish the Doppler signals produced by the movement of the heart wall and valve, and, further, Doppler signals originating from a diseased heart. They also developed a more coherent and complete recording of heart activity by recording Doppler signals simultaneously with the electrocardiogram and phonocardiogram on oscillograph paper. Information on valve movement - previously unavailable without invasive tools - and aspects linked to myocardial excitation were obtained and analysed [3]. At an international meeting in London held 3 years later (The 3rd International Conference on Medical Electronics), they named the above mentioned equipment Ultrasonic Doppler Cardiograph [4].

As part of his doctor of medical science thesis, Satomura (working with the neurologist Ziro Zaneko) used a newly built Doppler flowmeter to produce data on blood flow velocities in peripheral and extracranial brain supplying vessels using ultrasound. They proved that ultrasonic Doppler signals from arteries and veins can be recorded from the surface of the skin and pioneered transcutaneous flow analysis in systole and diastole in both normal and diseased blood vessels [5].

Another one of the group’s innovations was the “Ultrasonic Blood Rheograph” [6], which had been presented by Ziro Zaneko at the same 3rd International Conference on Medical Electronics. It was postulated that, while the Doppler flowmeter could not measure quantitative blood flow volume, it could qualitatively examine the characteristic rheological changes of blood flow in various clinical conditions.

In recognition of his new interest in medicine and full dedication to developing engineering tools for medical applications, Satomura was awarded the degree of Doctor in Medical Sciences in 1960.

Shigeo Satomura was the heart of a multidisciplinary team joining the expertise of engineers and physicians and was propelled by the university’s prestige and funding from industry.

Unfortunately, at the age of 41, in the midst of working to prove his ideas and to finish his ongoing research, this bright-minded and hard-working man passed away suddenly due to a subarachnoid haemorrhage.

After Satomura’s death, the Japanese team of physicists and physicians in Osaka continued on his path. Satomura considered that the Doppler signals were generated from turbulent flow in the blood vessels like the noise heard through a stethoscope, it was later clarified by Kanemasa Kato (Osaka 1962), that in fact the flow signals originated from reflections from the red blood cells moving simultaneously at different velocities. In addition, it was shown that the wave frequency shift was proportional to the velocity of the flow and that the magnitude of the voltage output corresponded to the number of red blood cells [7]. One of the early criticisms of the Doppler flowmeter, its inability to detect flow direction, was overcome by Kato and Izumi who developed in 1966 the first directional flow-meter [8].

Over the following years of early research, American (Dean Franklin, Donald W. Baker) and European teams (Peter Wells, Paul Peronneau) also joined the competition [9, 10]. Because Japanese reports were almost exclusively written in Japanese, the benefits of shared data were not used. On a parallel evolution in USA, Dean Franklin’s group at the University of Washington, Seattle also described a ultrasound flowmeter in 1959; this, unlike the one developed by Satomura, was an invasive device which was attached to the blood vessel wall [11]. They later reported transcutaneous Doppler flow detection as a non-invasive technique in 1966. Independently of the Osaka group, McLeod developed in 1967 the directional Doppler [11, 12].

The pulsed Doppler technique was developed almost simultaneously by Wells in the UK and Peronneau et al. in France in 1969, and in 1970 by Baker in the United States and Ohtsuki and Okujima in Japan [11, 13]. An important step toward spreading Doppler technique in clinical practice was represented by its combination with 2D imaging with the development of ultrasonic duplex echo-Doppler scanner by Frank Barber in 1974 [14]. Another breakthrough came when Holen and then Hatle demonstrated that hemodynamic data in patients with mitral stenosis and aortic stenosis respectively can be accurately determined with Doppler ultrasound by using the Bernoulli equation [15].

## Conclusions

Shigeo Satomura discoveries were the first medical applications of Doppler sonography and impressive technologic innovations have been continuing ever since. Over time, Doppler techniques became a key player in diagnostic ultrasound for hemodynamic assessment, replacing cardiac catheterization in many clinical settings.
